# Integrated Metagenomic and Metabolomic Analysis on Two Competing Mussels, *Mytella strigata* and *Perna viridis*, in China

**DOI:** 10.3390/ani14060918

**Published:** 2024-03-16

**Authors:** Chenxia Zuo, Peizhen Ma, Xiaojie Ma, Yi Zhu, Shaojing Yan, Zhen Zhang

**Affiliations:** 1 College of Life Sciences, Qingdao University, Qingdao 266071, China; zuochenxia@qdio.ac.cn; 2 Laboratory of Marine Organism Taxonomy & Phylogeny, Qingdao Key Laboratory of Marine Biodiversity and Conservation, Institute of Oceanology, Chinese Academy of Sciences, Qingdao 266071, China; maxj@qdio.ac.cn (X.M.); zhuyi@qdio.ac.cn (Y.Z.); yansj@qdio.ac.cn (S.Y.); 3 State Key Laboratory of Mariculture Biobreeding and Sustainable Goods, Yellow Sea Fisheries Research Institute, Chinese Academy of Fishery Sciences, Qingdao 266071, China; mapz@ysfri.ac.cn; 4 Key Laboratory of Sustainable Development of Marine Fisheries, Ministry of Agriculture and Rural Affairs, Yellow Sea Fisheries Research Institute, Chinese Academy of Fishery Sciences, Qingdao 266071, China; 5 University of Chinese Academy of Sciences, Beijing 100049, China

**Keywords:** biological invasion, intestinal microbiota, metabolite, enzyme activity

## Abstract

**Simple Summary:**

Biological invasion, particularly by the invasive mussel *Mytella strigata* (Hanley, 1843), has led to a significant decline in the population of native *Perna viridis* (Linnaeus, 1758) in western Pacific regions. This study compared the intestinal microbiota, metabolome, and key digestive enzymes of these two competing mussels to elucidate their differences from intestinal microbiota and metabolic points. The analysis revealed differences in the abundance of *Bacteroidota* related to carbohydrate degradation, as well as enriched metabolites and higher carbohydrase activities in *M. strigata* compared to *P. viridis*. These findings suggested that these differences might contribute to the adaptation of *M. strigata* to its new environment, providing valuable insights into the competition between these species for food resources.

**Abstract:**

Biological invasion is a primary direct driver of biodiversity loss. Recently, owing to exploitation competition with an invasive mussel, *Mytella strigata* (Hanley, 1843), there has been a drastic decrease in the population of native *Perna viridis* (Linnaeus, 1758) in several western Pacific regions. In the present study, intestinal microbiota, metabolome, and key digestive enzyme activities were compared between the two competing mussels, *M. strigata* and *P. viridis*, to elucidate the differences in intestinal microbiota and metabolic points. We observed that *Proteobacteria*, *Firmicutes*, and *Bacteroidota* were the three predominant bacterial phyla in the two species. The relative abundance of *Bacteroidota* related to carbohydrate-degrading ability was significantly higher in *M. strigata* than in *P. viridis*. Compared to *P. viridis*, different metabolites including maltose and trehalose were enriched in *M. strigata*. Lastly, higher carbohydrases activities of alpha-amylase, cellulase, and xylanase were observed in *M. strigata* than in *P. viridis*. These differences might play an important role in the adaptation process of *M. strigata* to the new environment. This study provides important basic knowledge for investigating the competition between *M. strigata* and *P. viridis* in terms of food resources utilization.

## 1. Introduction

Biological invasion is one of the greatest threats to native biodiversity and ecosystem integrity [1,2]. The typical ecological impacts include changes in physical conditions, preying, and competition with native species for space; this has resulted in the decrease in or extinction of native species and deleterious effects on ecosystem functions [3,4]. Competition between invasive and native species occurs via two primary processes: interference and/or exploitation [5]. In exploitation competition, invasive and native species share the same limited resources, including food, water, and space [6]. However, compared with native species, invaders can use resources more efficiently [7,8,9] and become dominant, converting the advances into higher growth rates and/or fecundity [10,11]. Furthermore, species interactions with invasive species may affect the native biota [12,13]. The increase in the number of new hosts provides opportunities for expanding the population of native parasites and increasing the infection levels of the original native hosts [14,15]. For example, the invasion of the Pacific oyster *Magallana gigas* (Thunberg, 1793) (Fam. Ostreidae) has contributed to the distribution and abundance of parasite infections in native mussels in the European Wadden Sea [16].

Many mussels are successful biological invaders and have benefited from their abilities of rapid growth, early maturity, high fecundity rate, strong adhesive force, and adaptability to different habitat conditions [2,4,17,18]. For example, the bivalve of the family Dreissenidae zebra mussel, *Dreissena polymorpha* (Pallas, 1771), a world-famous biological invader, is present in all European and North American regions [19,20]. This mussel has increased the regional extinction rates of freshwater mussels by 10-fold in North America and has been responsible for the extinction of native mussel populations [19]. *Xenostrobus securis* (Lamarck, 1819) (Fam. Mytilidae), another biologically and ecologically similar mussel, has caused a high resistance to water flow in tunnels, the corrosion of pipe walls, and the clogging of tunnels in South America [21,22]. *Mytella strigata* (Hanley, 1843) (Fam. Mytilidae), also known as charru mussel, is native to the Pacific and Atlantic coasts of tropical America [23]; however, it has successfully invaded Florida and the western Indo-Pacific region recently [23,24,25,26,27]. Its adaptation and survival ability at temperatures of 9 °C–31 °C in salinities of 2–40 has made it a successful invasive species; this mussel causes substantial losses to the aquaculture industry and decreases native biodiversity [28]. In oyster farms in the United States and the Philippines, *M. strigata* attached to farming facilities, competed with oysters for food, and decreased oyster production by 60–70% [24,29,30,31]. Furthermore, in farming ponds for clams and shrimps, the dense distribution of *M. strigata* at the bottom of the ponds has resulted in water deoxygenation, rapid bacterial proliferation, and serious production decline [26,32,33]. Owing to niche overlap and similar lifestyles, the invasion of *M. strigata* has drastically decreased the population of *Perna viridis* (Linnaeus, 1758) (Fam. Mytilidae) in Kerala’s coast of India and East Johor Strait of Singapore, even to the brink of extinction [23,28,33,34]. However, the mechanism underlying the exploitation competition between *M. strigata* and *P. viridis* for resources remains unelucidated. The intestinal microbiota is vital for food digestion and absorption and generating nutrients required for the growth and development of animal reservoirs [35,36]. Furthermore, the activities of digestive enzymes also indicate the ability of the intestine to digest and absorb nutrients [37]. As a result, studying the gut microbiome, metabolites, and digestive enzymes activities may explain the differences between *M. strigata* and *P. viridis* in terms of resource utilization.

In the present study, samples of both *M. strigata* and *P. viridis* were collected from China, where the invasion of *M. strigata* is in the establishment and spread stages and poses a great threat to local *P. viridis* [38,39,40]. The intestinal microbiota, metabolites, and digestive enzyme activity of both species were compared. Our study aims were as follows: (1) to identify the composition of the intestinal microbes in *M. strigata* and *P. viridis*; (2) to search for differences in the metabolic pathways of *M. strigata* compared with *P. viridis*; and (3) to compare the activities of carbohydrases between *M. strigata* and *P. viridis*.

## 2. Materials and Methods

### 2.1. Sample Collection

Both *M. strigata* and *P. viridis* were collected from Donghai Island (20°55′53″ N, 110°31′43″ E), Zhanjiang, Guangdong Province, China, on 31 March 2023. Sampling was limited to the same reef to ensure that both species have the same food source. Subterminal umbos, external shell color, color patterns, and 3–4 (as many as 7) teeth in the anterior ventral region of the valves were used as parameters to identify and differentiate *M. strigata* from *P. viridis* [23,26,39,40]. The average shell lengths of *M. strigata* and *P. viridis* were 28.32 ± 2.21 and 27.48 ± 5.19 mm, respectively. The intestines of both species were immediately collected. Intestinal tissue from approximately 100 individuals constituted one replicate to ensure the weight was >1 g. Three replicates were set up for each species. All intestinal samples were flash frozen and stored in liquid nitrogen.

### 2.2. Metagenomic Sequencing and Analysis

The cetyltrimethylammonium bromide method was used to extract the total genomic DNA of the microbes in the intestine samples. Then, the degree of DNA degradation and potential contamination were quantified on 1% agarose gels. The Qubit^®^ dsDNA Assay Kit in the Qubit^®^ 2.0 Fluorometer (Life Technologies, Carlsbad, CA, USA) was used to measure DNA concentration (μg/mL). The NEBNext^®^ Ultra™ DNA Library Prep Kit was used according to the manufacturer’s recommendations to generate sequencing libraries. Index codes were added to attribute sequences to each sample. The Illumina NovaSeq platform (Illumina, San Diego, CA, USA) at Wuhan Metware Biotechnology Co., Ltd. (Wuhan, China) was used to sequence the libraries. Approximately 3 Gbp of 150 bp paired-end reads per sample were generated.

After sequencing, Fastp software (https://github.com/OpenGene/fastp, accessed on 5 May 2023) was used to perform the quality control of raw data with default parameters [41]. Bowtie 2 (Version 2.3.4, parameters: end-to-end, sensitive, I 200, x 400) was used to filter the reads that may be from the host [42,43]. After pretreatment, clean data were obtained. MEGAHIT 1.2.9 (parameters: --k-list 21, 41, 61, 81, 91, --min-contig-len 500) was used to perform metagenomic assembly analysis of the clean reads [44]. Based on the contigs (≥500 bp) of each sample and the mixed assembly, MetaGeneMark 3.38 was used with the default parameters to predict the open reading frames [45,46]. CD-HIT 4.8.1 software using the parameters c 0.95, g 0, AS 0.9, g 1, d 0, and identity ≥ 95% was used to annotate the unigenes [47,48]. For taxonomic annotation, DIAMOND 0.7.9.58 software (BLastP, EVALue ≤ 1 × 10^−5^) was used to align the unigenes to the NR 2022.05 database [49]. The lowest common ancestor algorithm in MEGAN 4 software was used to obtain the final annotation information of the species [50]. R 4.2.0 was used to conduct principal coordinate analysis (PCoA) to further evaluate dissimilarities in microorganic composition based on the Bray–Curtis distance. For functional annotation, unigenes were matched to the KEGG 2022.05 [51,52] and CAZy 2022.05 [53] databases using DIAMOND 0.7.9.58 software (BLastP, EVALue ≤ 1 × 10^−5^). KEGG level 1 contains broad biological categories and level 2 contains more specific pathways within each category, representing distinct biological processes or functions. KEGG level 3 contains further subdivisions within pathways and level 4 contains individual genes or enzymes involved in specific biological pathways or modules. QIIME 1.9.1 was used to calculate the alpha diversities. Metastats software (http://metastats.cbcb.umd.edu/, accessed on 5 May 2023) was used to perform differential analysis (taxa and functional modules) [54]. R 4.2.0 was used to perform line discriminant analysis (LDA) to identify the differentially represented features (taxa and functional modules) between the two groups. Python 3.8.12 was used to perform LDA effect size (LEfSe).

### 2.3. Intestinal Metabolomic Analysis

First, samples were ground with liquid nitrogen. Then, a 400 μL solution (methanol– water = 7:3, *V*/*V*) containing the internal standard was added to 20 mg of the ground sample and shaken at 2348× *g* for 5 min. After placing the samples on ice for 15 min, they were centrifuged at 13,523× *g* for 10 min at 4 °C. The supernatant (300 μL) was collected and placed at −20 °C for 30 min. Thereafter, the samples were centrifuged at 13,523× *g* for 3 min (4 °C). A 200 μL aliquot of the supernatant was transferred for liquid chromatography–mass spectrometry (LC–MS) analysis. The Acquity ultra-performance liquid chromatography (UPLC) system (Waters, Milford, MA, USA) was used to quantify the metabolites. The BEH C18 column (1.8 µm, 2.1 mm × 100 mm, Waters) was used for separation. Mobile phase A comprised 0.1% formic acid in water, and mobile phase B comprised 0.1% formic acid in acetonitrile. The column was maintained at 40 °C. The flow rate was 0.4 mL·min^−1^, and the injection volume was 2 μL. The column was eluted with 5% mobile phase B at 0 min, followed by a linear gradient to 90% mobile phase B over 11 min, held for 60 s, and then returned to 5% mobile phase B within 6 s, held for 114 s, and then rapidly returned to the initial conditions. The information-dependent acquisition mode was used to acquire data using Analyst TF 1.7.1 Software (Sciex, Concord, ON, Canada). The source parameters were set as follows: ion source gas 1, 50 psi; ion source gas 2, 50 psi; curtain gas, 35 psi; temperature, 550 °C or 450 °C; declustering potential, 60 V or −60 V in the positive or negative mode, respectively; and ion spray voltage floating, 5000 V or −4000 V in the positive or negative mode, respectively. The time-of-flight MS scan parameters were set as follows: mass range, 50–1000 Da; accumulation time, 200 ms; and dynamic background subtract, on. The product ion scan parameters were set as follows: mass range, 25–1000 Da; accumulation time, 40 ms; collision energy, 30 or −30 V in the positive or negative mode, respectively; collision energy spread, 15; resolution, UNIT; charge state, 1 to 1; intensity, 100 cps; exclude isotopes within 4 Da; mass tolerance, 50 mDa; and maximum number of candidate ions to monitor per cycle, 12.

ProteoWizard software (https://proteowizard.sourceforge.io/, accessed on 5 May 2023) was used to convert the original data file acquired via LC–MS analysis into the mzML format [55]. The XCMS program was used to perform peak extraction, peak alignment, and retention time correction. The “SVR” method was used to correct the peak area. The peaks with a detection rate of <50% in each sample group were removed. Then, metabolites were identified by searching the laboratory’s self-built database, integrated public database, AI database, and metDNA. The statistical function prcomp in R 4.2.0 was used to perform unsupervised principal component analysis (PCA). For two-group analysis, differential metabolites were determined as follows: variable importance in projection (VIP) > 1 and *p*-value < 0.05 (Student’s *t*-test). Orthogonal partial least squares discriminant analysis (OPLS-DA) was used to extract the VIP values. It also contains score plots and permutation plots, which were generated using the R package MetaboAnalystR 1.0.1. The data were log transformed (log_2_) and mean centered before performing OPLS-DA. To avoid overfitting, a permutation test (200 permutations) was performed. The identified metabolites were annotated using the KEGG Compound database (http://www.kegg.jp/kegg/compound/, accessed on 5 May 2023). The annotated metabolites were then mapped to the KEGG Pathway database (http://www.kegg.jp/kegg/pathway.html, accessed on 17 May 2023). Significantly enriched pathways were identified using the *p*-values for a given list of metabolites with the hypergeometric test.

### 2.4. Activities of Carbohydrases Assay and Statistical Analysis

The activity of alpha-amylase, cellulase, and xylanase were measured by an alpha-amylase (AMS) ELISA assay kit for fish, cellulase ELISA assay kit for fish, and xylanase ELISA assay kit for fish, respectively (Jiangsu Maisha Industries Co., Ltd., Yancheng, China), according to the manufacturer’s instructions. All values were presented as mean ± standard deviation. Student’s *t*-test was used to perform statistical analysis of the quantitative multiple group comparisons. Spearman’s correlation analysis was performed to determine the correlation between the significantly different microbiomes and significantly different metabolites. R 4.2.0 was used to generate the graphs. Results with a *p*-value of <0.05 were considered statistically significant.

## 3. Results

### 3.1. Metagenomic Analysis

In total, 78,559 and 84,898 unique unigenes were screened in the gut microbiota of the *M. strigata* and *P. viridis* groups, respectively, with 2206 unigenes shared by both groups (Figure S1a). Alpha diversity analysis revealed that the coverage indexes of both groups were >99%. The Shannon index, which represented microbiota diversity of the *P. viridis* group, was 14.35; this was higher than that of the *M. strigata* group (13.99, *p* < 0.05). However, no significant difference in microbiota richness, as represented by the ACE and Chao1 indexes, was observed between the groups (Table 1). Furthermore, PCoA analysis revealed that *M. strigata* and *P. viridis* were successfully separated (R = 1, *p* = 0.10), with 95.85% and 2.86% of the variations explained by the principal components PC1 and PC2, respectively (Figure S1b).

**Figure 1:**
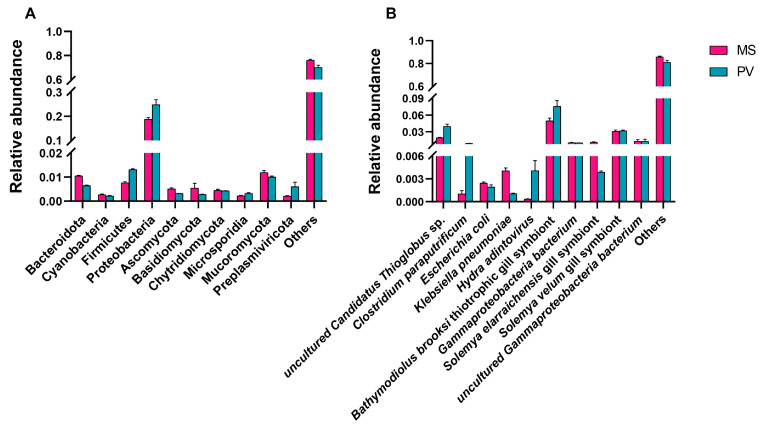
(**A**) Relative abundances of microorganic phyla in *Mytella strigata* and *Perna viridis*. (**B**) Relative abundances of microorganic species in *M. strigata* and *P. viridis*. MS represents *M. strigata* group. PV represents *P. viridis* group.

**Figure 2:**
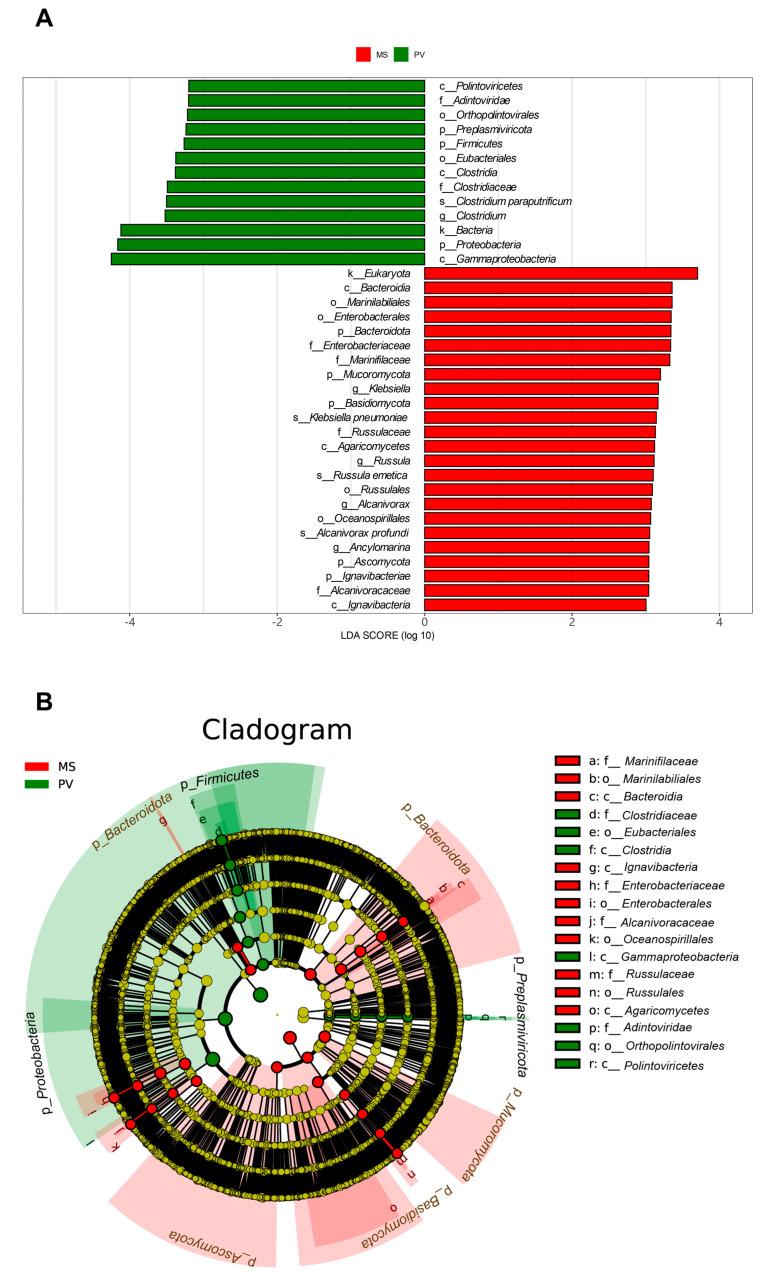
(**A**) LDA scores of the differentially abundant microorganic taxa between *Mytella strigata* and *Perna viridis* (LDA > 3). (**B**) Cladograms indicating differences in the microorganic taxa between *M. strigata* and *P. viridis*. MS represents *M. strigata* group. PV represents *P. viridis* group.

At the phylum level, *Proteobacteria*, *Firmicutes*, and *Bacteroidota* were the three predominant phyla in both groups. The relative abundances of *Proteobacteria* (24.83%) and *Firmicutes* (1.31%) were significantly higher in *P. viridis* than in *M. strigata* (18.71% and 0.76%, respectively, *p* < 0.05); in contrast, the relative abundance of *Bacteroidota* (1.05%) was higher in *M. strigata* than in *P. viridis* (0.64%, *p* < 0.05) (Figure 1A). The relative abundances of *Microsporidia* (0.32%) and *Preplasmiviricota* (0.60%) were significantly higher in *P. viridis* than in *M. strigata* (0.22%, 0.20%, respectively, *p* < 0.05). In both groups, at the species level, unclassified *Bathymodiolus brooksi* thiotrophic gill symbiont, *Solemya velum* gill symbiont, and uncultured *Candidatus Thioglobus* sp. were predominant in the intestinal microbiota. The relative abundances of *Bathymodiolus brooksi* thiotrophic gill symbiont (7.56%) and *Candidatus Thioglobus* sp. (3.96%) were higher in *P. viridis* than in *M. strigata* (4.95% and 1.91%, respectively, *p* < 0.05) (Figure 1B). LDA coupled with LEfSe analysis identified 24 and 13 biomarkers in the *M. strigata* and *P. viridis* groups, respectively, including *Proteobacteria*, *Bacteroidota*, *Clostridium paraputrificum*, *Klebsiella pneumoniae*, *Russula emetica*, and *Alcanivorax profundi* (Figure 2A,B).

**Figure 3:**
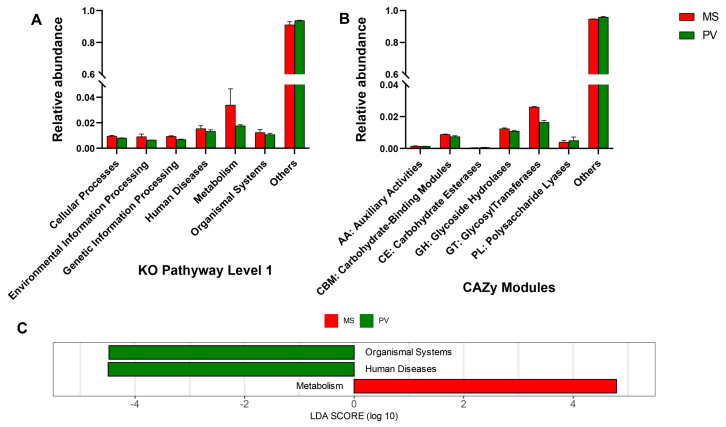
(**A**) Relative abundance of the functional predictions based on KEGG pathway level 1 in *Mytella strigata* and *Perna viridis*. (**B**) Relative abundance of the functional predictions based on the CAZy database in *M. strigata* and *P. viridis*. (**C**) LDA of the gut microbial function based on KEGG pathway level 1 in *M. strigata* and *P. viridis*. MS represented *M. strigata* group. PV represented *P. viridis* group.

The functional annotation of the gut microbiota using the KEGG databases revealed that the gut microbiota of both groups had different functional compositions. The relative abundance of metabolic pathways was the highest in both groups (3.39% in *M. strigata* and 1.76% in *P. viridis*), compared with that of the other pathways. Furthermore, cellular processes and genetic information processing pathways were significantly higher in *M. strigata* (0.95% and 0.93%, respectively) than in *P. viridis* (0.80% and 0.68%, respectively, *p* < 0.05) (Figure 3A). In addition, the annotation results based on the CAZy database indicated that glucosyltransferase enzymes were predominant in both groups (2.60% in *M. strigata* and 1.64% in *P. viridis*). The relative abundance of carbohydrate-binding modules (0.87%) and glycoside hydrolases (1.23%) was higher in *M. strigata* than in *P. viridis* (0.74% and 1.09%, respectively, *p* < 0.05) (Figure 3B). Compared with *P. viridis*, the LDA scores of the KEGG function in *M. strigata* confirmed a significant enrichment of the metabolic pathway (Figure 3C). Furthermore, level 2 in the KEGG database revealed that the metabolic subfunctions of genetic information processing, cellular processes, and organismal systems were significantly higher in the *M. strigata* group than in the *P. viridis* group, including glycan biosynthesis and metabolism, terpenoid and polyketide metabolism, and the translation of genetic information (Table 2).

### 3.2. Intestinal Metabolomic Analysis

Nontargeted metabolomic analysis based on the UPLC–MS method revealed that the metabolites in the intestinal homeostasis of the two groups were well separated, with 43.64% and 26.18% of variation explained by the principal components PC1 and PC2, respectively, as revealed by the PCA plot (Figure S2a). The OPLS-DA scores revealed that *M. strigata* and *P. viridis* were dispersed in two different regions (Figure S2b). The goodness-of-fit values and predictive ability values (*M. strigata* vs. *P. viridis*: R2X = 0.657, R2Y = 0.997, Q2 = 0.902, and *p* < 0.005) revealed that the OPLS-DA model possesses a satisfactory fit with good predictive power (Figure S2c).

**Figure 4:**
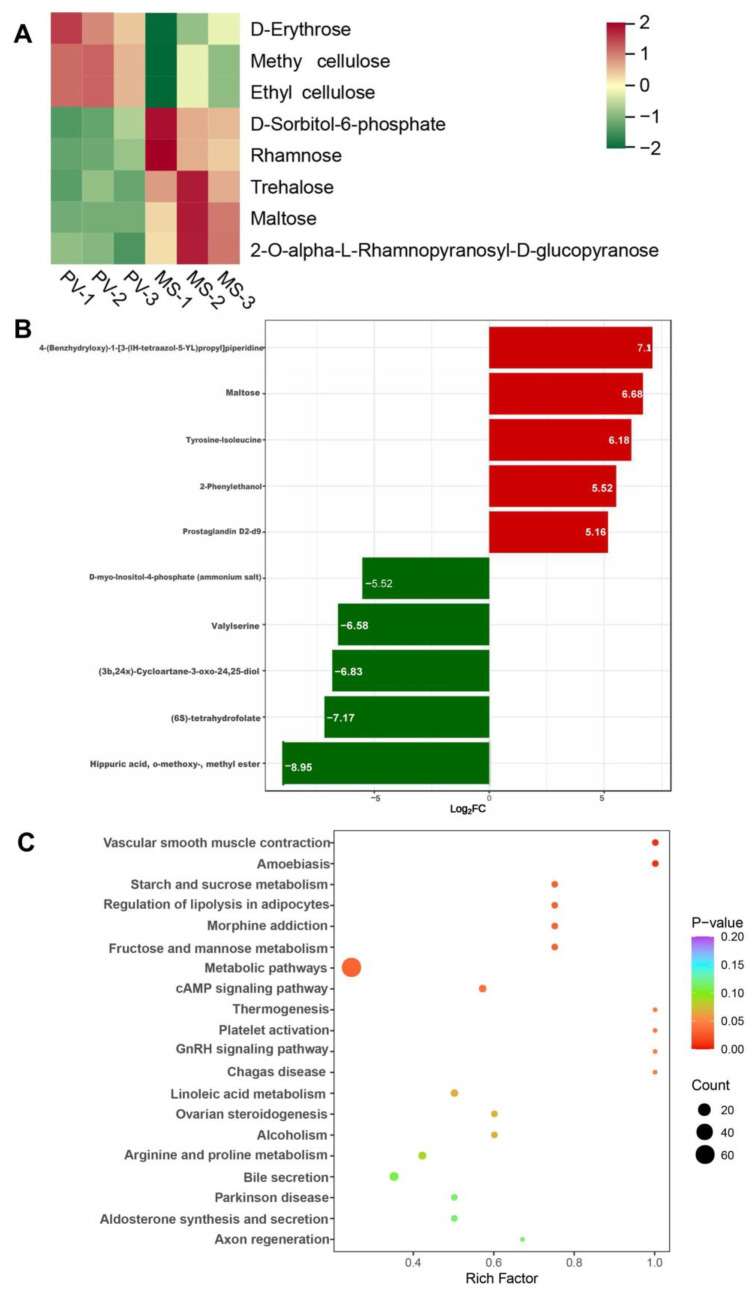
(**A**) Heatmap showing the differentially expressed metabolites between *Mytella strigata* and *Perna viridis* in terms of the classes of carbohydrates and their metabolites. (**B**) Bar chart showing the fold changes of the metabolites in *M. strigata* and *P. viridis*. (**C**) KEGG pathway enrichment plot for the differential metabolites between *M. strigata* and *P. viridis*. MS represented *M. strigata* group. PV represented *P. viridis* group.

Using VIP > 1 and Student’s *t*-test with a *p*-value < 0.05, 399 differential metabolites (168 upregulated and 231 downregulated) were identified in the *M. strigata* versus *P. viridis* groups (Figure S3). A heatmap of the classes of the carbohydrates and their metabolites demonstrated the enrichment of 2-*O*-alpha-l-rhamnopyranosyl-d-glucopyranose, maltose, trehalose, rhamnose, and d-sorbitol-6-phosphate in the *M. strigata* group (Figure 4A). The metabolites 4-benzhydryloxy-1-(3-(1*H*-tetrazol-5-yl-)-propyl) piperidine, maltose, tyrosine-isoleucine, 2-phenylethanol, prostaglandin D2-d9, and D-ribose were significantly enriched in the *M. strigata* group compared with the *P. viridis* group (Figure 4B). In addition, the differential metabolites were matched with the KEGG database; the results revealed that these metabolites represented key metabolic pathways, including metabolic pathways, fructose and mannose metabolism, and starch and sucrose metabolism (Figure 4C).

### 3.3. Potential Correlations between the Gut Microbiota and Metabolites

**Figure 5:**
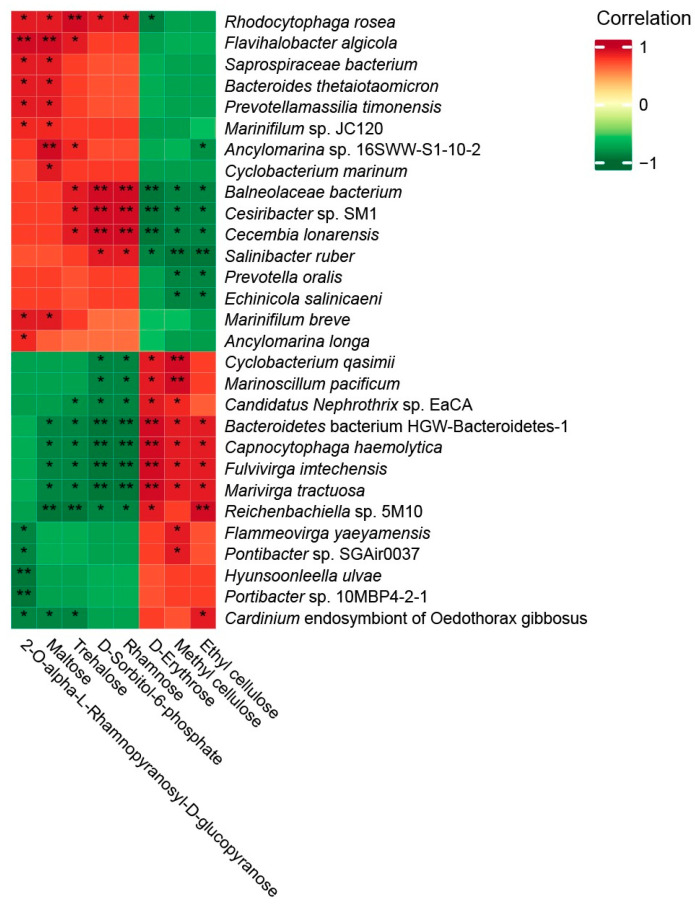
Heatmap of Spearman’s correlation coefficient matrix for the significantly different intestinal microbiota and metabolites (* *p* < 0.05 and ** *p* < 0.01).

The Spearman’s correlation coefficient matrix heatmap displayed the correlation between 29 different microbiotas of *Bacteroidota* and 8 carbohydrates (Figure 5). Among them, *Rhodocytophaga rosea* was positively correlated with 2-O-alpha-L-rhamnopyranosyl-D-glucopyranose, maltose, trehalose, rhamnose, and D-sorbitol-6-phosphate (*r* > 0.8, *p* < 0.05). Furthermore, *Flavihalobacter algicola* and *Ancylomarina* sp. 16SWW-S1-10-2 were positively correlated with maltose and trehalose (*r* > 0.8, *p* < 0.05). Moreover, *Balneolaceae bacterium*, *Cesiribacter* sp. SM1, and *Cecembia lonarensis* were positively correlated with rhamnose, D-sorbitol-6-phosphate, and trehalose but negatively correlated with ethyl cellulose, methyl cellulose, and D-erythrose (*r* > 0.8, *p* < 0.05).

### 3.4. Activities of Carbohydrases

**Figure 6:**
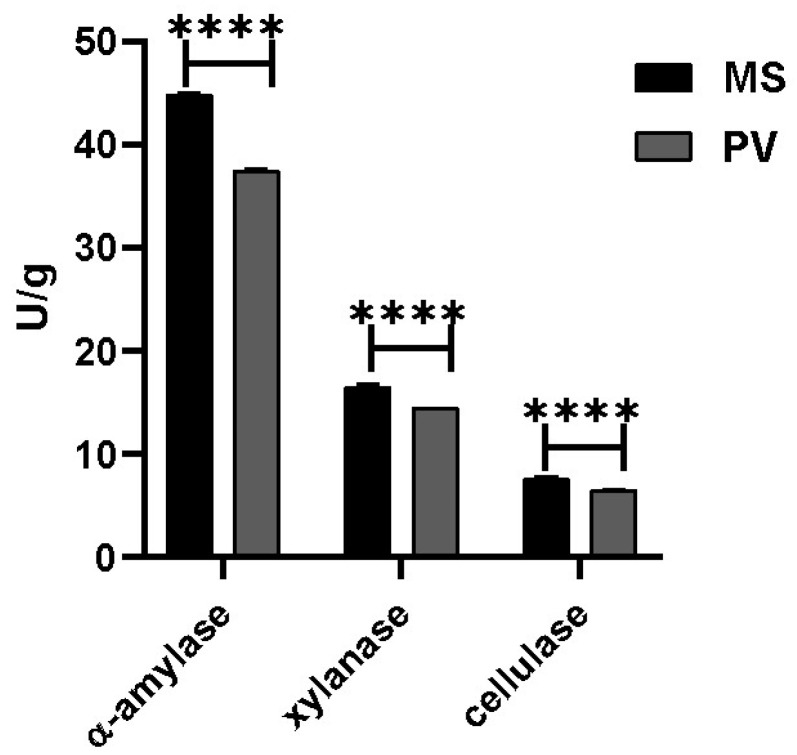
Specific activities (U/g) of alpha-amylase, cellulase, and xylanase in *Mytella strigata* group and *Perna viridis* group. MS represents *M. strigata* group. PV represents *P. viridis* group. **** *p* < 0.0001.

Carbohydrase activities of the two groups significantly differed. Specific activities of alpha-amylase, cellulase and xylanase were 44.8 U/g, 16.43 U/g, and 7.59 U/g, respectively, in the *M. strigata* group and 37.44 U/g, 14.38 U/g, and 6.41 U/g, respectively, in the *P. viridis* group. The specific activities of the three carbohydrases in the *M. strigata* group were significantly higher than in the *P. viridis* group (*p* < 0.0001) (Figure 6).

## 4. Discussion

Considering that the intestinal microbiota of animals is significantly affected by diet, living environment, and other factors [56,57], the two species were sampled from the same environment to avoid other disruptive factors. They were close to each other on the same reef, with their byssus entwined (Figure S4). Bivalves can selectively consume particles with diverse range sizes, and mussels can access a wider size spectrum of food particles compared to oysters [58]. For mussels, individuals of *Perna canaliculus* (Gmelin, 1791) (Fam. Mytilidae) with 0.3 mm and 1.0 mm shell length were unable to selectively capture particles, while larger juveniles (7.0 mm shell length) actively captured particles [59]. *M. strigata* and *P. viridis* had similar shell length in this study. Moreover, *Rhodobacteraceae* were represented as constituents of larger particle sizes [60] and they presented in the *M. strigata* group and *P. viridis* group with relative abundances of 0.0085% and 0.0095%, respectively. Thus, we hypothesized that *M. strigata* and *P. viridis* have comparative size spectra of food particles.

Intestinal metagenomic analysis revealed that the relative abundance of *Bacteroidota* related to carbohydrate-degrading ability was significantly higher in *M. strigata* than in *P. viridis* and metabolomic analysis indicated that different metabolites including maltose and trehalose were enriched in *M. strigata*. Furthermore, higher activities of carbohydrases were observed in *M. strigata* than in *P. viridis*. However, the variations between *M. strigata* and *P. viridis* might also be due to genetic factors or differences in filtering efficiency [61].

### 4.1. Gut Microbiota Is Associated with Carbohydrate Degradation in M. strigata

*Proteobacteria*, *Firmicutes*, and *Bacteroidota* were the three predominant intestinal phyla in *M. strigata* and *P. viridis*. *Proteobacteria* comprises carbohydrate-fermenting bacteria and can enhance the ability to use complex carbohydrates [62], and *Firmicutes* can help harvest energy [57]. *Bacteroidota* plays a vital role in the degradation of complex molecules, including polysaccharides, which is important for optimal energy uptake in the host [63]. The function of *Bacteroidota* to degrade glycan is often accomplished by the polysaccharide utilization locus (PUL) gene cluster and starch utilization system (Sus) [64]. After primary degradation, monosaccharides can be consumed for pyruvate and subsequent ATP production by the Embden–Meyerhof–Parnas (EMP) pathway, Entner–Doudorof (ED) pathway, or pentose phosphate (PP) pathway. *Bacteroidota* has complete EMP and PP cycles and can encode the key enzyme in the ED pathway (KDPG aldolase) [65,66]. The high abundance of *Bacteroidota* may suggest the high ability to degrade polysaccharides. For functional prediction, Figure 3A,B and Table 2 demonstrate that glycan biosynthesis and metabolism and terpenoid and polyketide metabolism based on the KEGG pathways and carbohydrate-binding modules and glycoside hydrolases based on the CAZy database are obviously enriched in the *M. strigata* group; this suggests that the gut microbiota of *M. strigata* can uptake nutrition efficiently. The results of taxonomic and functional annotation showed a high percentage of “Others”. There could be two reasons: (1) samples included a large number of unknown organisms, not included in the databases; (2) “others” were known organisms, but the databases covered limited information.

### 4.2. Differences in Carbohydrate and Organismal Metabolism between M. strigata and P. viridis

2-*O*-Alpha-l-rhamnopyranosyl-d-glucopyranose, maltose, trehalose, rhamnose, and d-sorbitol-6-phosphate were the enriched carbohydrates in *M. strigata*. Among them, maltose and trehalose are disaccharides that serve as energy sources [67,68] and are abundant in phytoplanktons such as Chlorella and microalga [69,70]. These two metabolites were enriched in the *M. strigata* group, indicating a better carbohydrate-degrading ability compared with the *P. viridis* group. Improved carbohydrate digestion can increase glucose release for host absorption [71]. Moreover, the enrichment of the pathways associated with fructose and mannose metabolism and starch and sucrose metabolism revealed differences in carbohydrate metabolism in the *M. strigata* and *P. viridis* groups. In addition, organismal metabolism in *M. strigata* was promoted by several differential metabolites, whose contents were several times higher than those in *P. viridis*. As an unambiguously assigned neurochemical [72], tyrosine and its subsequent metabolites can decrease reactive oxygen species production. On the other hand, the upregulation of tyrosine metabolism may play a positive role in decreasing oxidative damage in the intestines [73]. Furthermore, tyrosine upregulation may be an adaptation to promote metabolism in organisms [36]. 2-Phenylethanol can increase cellular NAD(P)H levels and the expression of TCA (tricarboxylic acid) cycle-related genes, including acnB, ilvB, sdhA, and citH [74]. Moreover, d-ribose, a five-carbon furanose, possesses strong reducing properties [75,76,77] and is ubiquitous. It plays a vital role in organismal growth and development because it is an essential component of RNA, nucleotides, B vitamins, and several coenzymes [78,79]. Collectively, these differential metabolites may explain the differences between *M. strigata* and *P. viridis* in interspecies competition.

### 4.3. Differences in Degraded Carbohydrates between M. strigata and P. viridis

Food digestion is a critical process for animals because it supplies the nutrients needed for all biological functions [80]. Intestinal digestive enzymes play a key role in food hydrolysis and the main carbohydrase enzymes of mussels include amylase, cellulase, laminarinase, and xylanase [81]. Amylase produces glucose, maltose and maltotriose from starch and the cellulase produces glucose and cellobiose from carboxymethyl cellulose [82]. Xylanase possesses the capability to hydrolyse xylan, which is the second most abundant structural polysaccharide in plant cell walls [83]. In this study, higher activities of amylase, cellulase, and xylanase in the *M. strigata* group than in the *P. viridis* group may imply a greater potential to degrade the carbohydrates of *M. strigata*. Intestinal microbiota can influence the production of some digestive enzymes to some extent [36]. However, whether the increase in the three carbohydrases activities was influenced by the intestinal microbiota needs to be further investigated.

## 5. Conclusions

In the present study, metagenomic, metabolomic, carbohydrases analyses clarified the differences between *M. strigata* and *P. viridis*. Compared with *P. viridis*, the relative abundance of *Bacteroidota* related to carbohydrate-degrading ability was higher in *M. strigata*. Different metabolites including maltose and trehalose were enriched in *M. strigata*. Higher carbohydrase activities of alpha-amylase, cellulase, and xylanase were observed in *M. strigata* compared to *P. viridis*. These variations may favor the adaptation of *M. strigata* to new environments and are significant for understanding interspecific competition between *M. strigata* and *P. viridis*. What is noteworthy is that the observed differences between *M. strigata* and *P. viridis* are restricted to the area studied, irrespective of their validity in other regions of China under different environmental setups.

## Figures and Tables

**Table 1:** Alpha diversity indexes of *Mytella strigata* and *Perna viridis*.

Group	Observed Unigenes	Coverage	Shannon *	Simpson	ACE	Chao1
*M. strigata*	57,122	1.00	13.99	1.00	57,211.65	57,170.95
*P. viridis*	63,041	1.00	14.35	1.00	63,241.71	63,139.14

* *p* < 0.05, Student’s *t*-test.

**Table 2:** Metastats statistical results of the relative abundance of *Mytella strigata* and *Perna viridis* based on KEGG pathway level 2.

		Relative Abundance (%)		*p*
Level 1	Level 2	*M. strigata*	*P. viridis*	
Metabolism	Glycan biosynthesis and metabolism	0.2756 ± 0.0299	0.1693 ± 0.0061	0.0037 *
	Metabolism of terpenoids and polyketides	0.2633 ± 0.0062	0.1565 ± 0.0368	0.0077 *
Cellular processes	Cellular community—prokaryotes	0.1282 ± 0.0066	0.1520 ± 0.0037	0.0055 *
	Transport and catabolism	0.4731 ± 0.0264	0.3824 ± 0.0333	0.0092 *
Genetic information processing	Folding, sorting, and degradation	0.3229 ± 0.0209	0.2444 ± 0.0150	0.0073 *
	Translation	0.2498 ± 0.0225	0.1635 ± 0.0424	0.0246 *
Organismal systems	Aging	0.0691 ± 0.0023	0.0743 ± 0.0029	0.0467 *
	Nervous system	0.2067 ± 0.0286	0.0983 ± 0.0055	0.0031 *
Human diseases	Infectious disease: bacterial	0.3117 ± 0.0485	0.4842 ± 0.0834	0.0252 *

Data are represented as mean ± standard deviation. * *p* < 0.05.

## Data Availability

The NCBI database SRA accession number for the raw high-throughput sequencing data is PRJNA1022301. The metabolome data have been deposited to the EMBL-EBI MetaboLights database and the accession number is MTBLS8684.

## Supplementary Materials

The following supporting information can be downloaded at: https://www.mdpi.com/article/10.3390/ani14060918/s1, Figure S1: (a) Venn diagram of the observed unigenes in the *Mytella strigata* (*M. strigata*) and *Perna viridis* (*P. viridis*) groups. (b) Principal Coordinate analysis (PCoA) of the microbiotas in *M. strigata* and *P. viridis* was generated by the Bray–Curtis distance based on phylum level; Figure S2: (a) PCA scatter plot of the metabolite profile in the *Mytella strigata* (*M. strigata*) group and the *Perna viridis* (*P. viridis*) group. The *M. strigata* group was separated from the *P. viridis* group. (b) OPLS-DA analyses of the metabolite profile in the *M. strigata* and *P. viridis* groups. (c) OPLS-DA model test chart; Figure S3: Volcano Plot showing differential metabolites between *Mytella strigata* and *Perna viridis*; Figure S4: *Mytella strigata* (red arrow) and *Perna viridis* (green arrow) co-attached to the same reef.
