# Supplementary material for: Integrated Metagenomic and Metabolomic Analysis on Two Competing Mussels, Mytella strigata and Perna viridis, in China
Source: Animals (Basel). 2024 Mar 16;14(6):918. doi: 10.3390/ani14060918 (PMC10967425; doi:10.3390/ani14060918)
Supplement: Supplementary file 1 [file animals-14-00918-s001.zip › animals-2830601-supplementary.pdf]

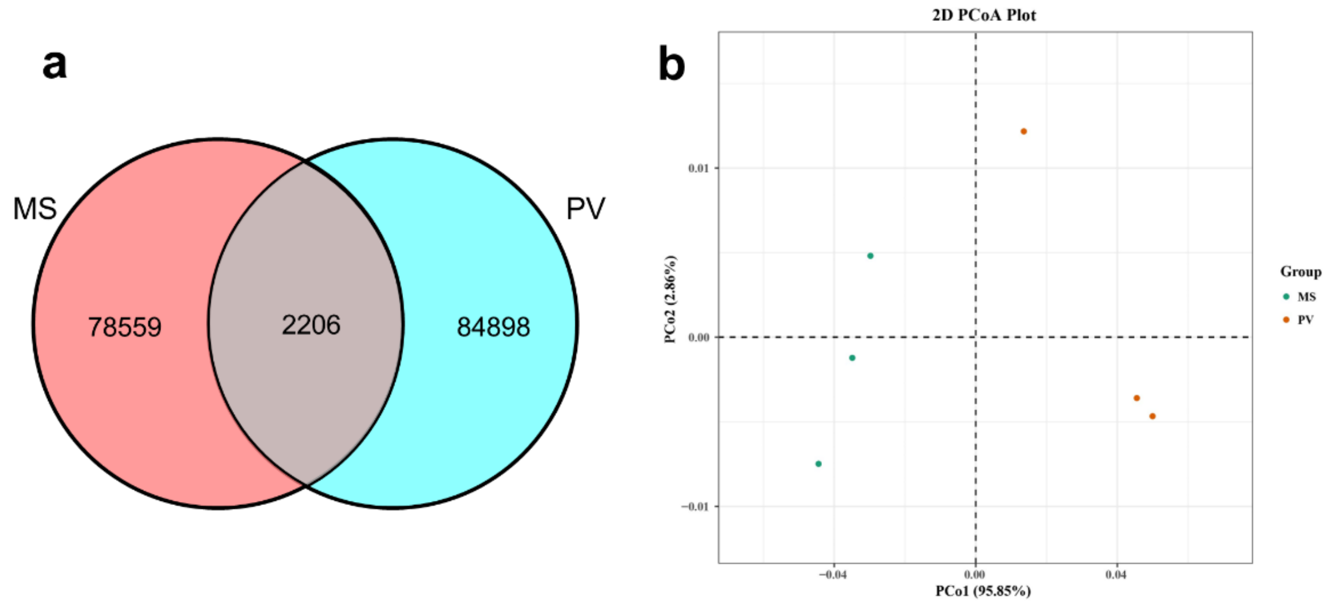

Figure S1. (a) Venn diagram of the observed Unigenes in *Mytella strigata* (*M. strigata*) and *Perna viridis* (*P. viridis*). (b) Principal Coordinate analysis (PCoA) of the microbiotas in *M. strigata* and *P. viridis* was generated of the Bray-Curtis distance based on phylum level. MS represented *M. strigata* group. PV represented *P. viridis* group.

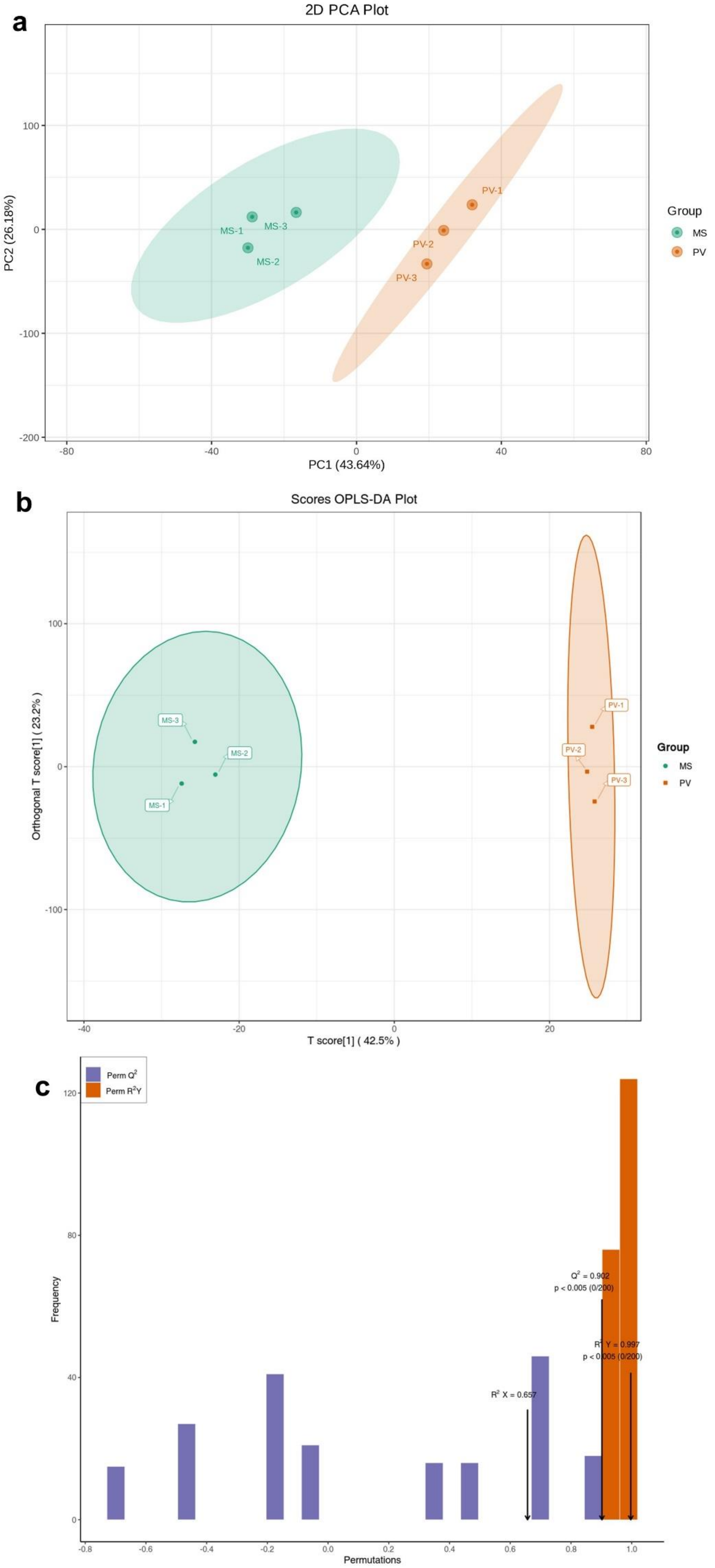
a
2D PCA Plot
PC1 (43.64%)
PC2 (26.18%)
Group
MS
PV
MS-1
MS-2
MS-3
PV-1
PV-2
PV-3
b
Scores OPLS-DA Plot
T score[1] (42.5%)
Orthogonal T score[1] (23.2%)
Group
MS
PV
MS-1
MS-2
MS-3
PV-1
PV-2
PV-3
c
Perm Q²
Perm R²Y
Frequency
Permutations
Q² = 0.902
p < 0.005 (0/200)
R² Y = 0.997
p < 0.005 (0/200)
R² X = 0.657

Figure S2. (a) PCA scatter plot of the metabolite profile in *Mytella strigata* (*M. strigata*) and *Perna viridis* (*P. viridis*). *M. strigata* group was separated from *P. viridis* group. (b) OPLS-DA analyses of the metabolite profile in *M. strigata* and *P. viridis* groups. (c) OPLS-DA model test chart showed good discrimination between the *M. strigata* and *P. viridis* groups, R2X = 0.657, R2Y = 0.997, Q2 = 0.902, $p < 0.005$. MS represented *M. strigata* group. PV represented *P. viridis* group.

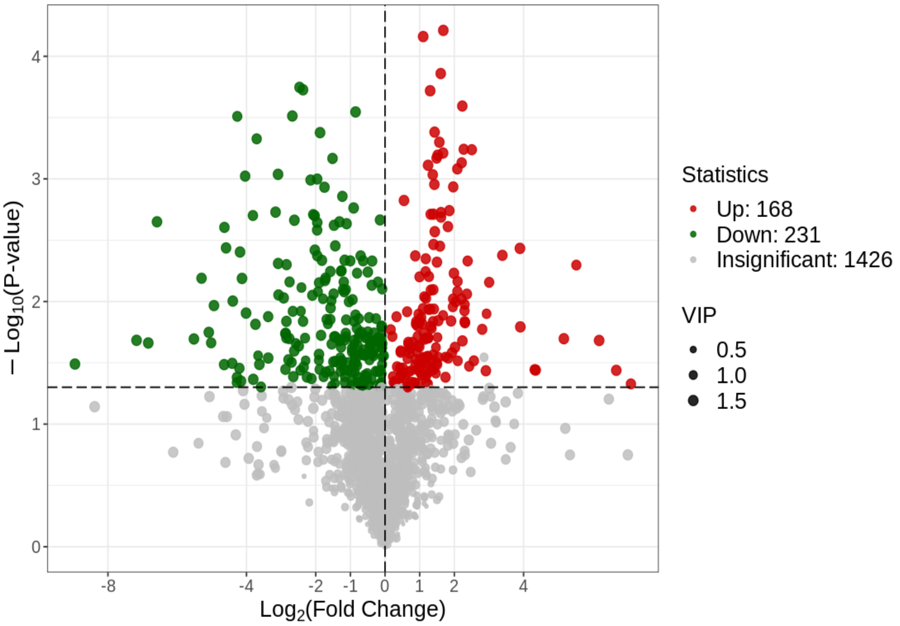

Figure S3. Volcano Plot showed differential metabolites between *Mytella strigata* and *Perna viridis*.

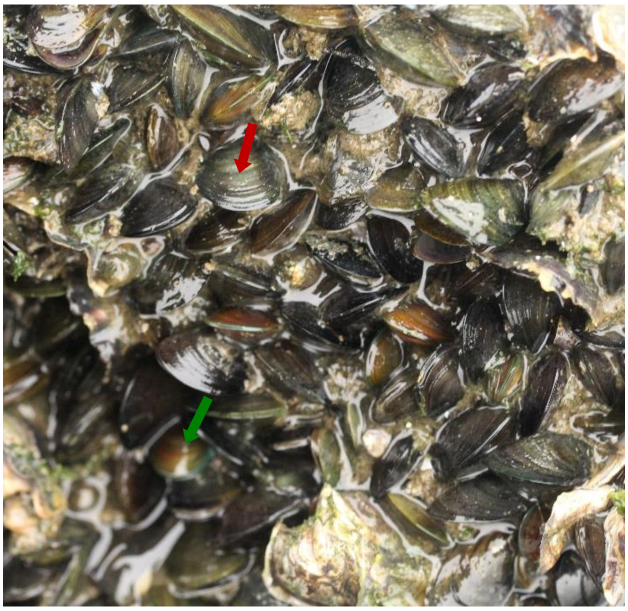

Figure S4. *Mytella strigata* (red arrow) and *Perna viridis* (green arrow) co-attached to the same reef.
